# Engineered exosomes as a prospective therapy for diabetic foot ulcers

**DOI:** 10.1093/burnst/tkae023

**Published:** 2024-07-18

**Authors:** Lifei Guo, Dan Xiao, Helin Xing, Guodong Yang, Xuekang Yang

**Affiliations:** Department of Burns and Cutaneous Surgery, Xijing Hospital, Fourth Military Medical University, Chang-Le Xi Street #127, Xi'an 710032, China; The State Laboratory of Cancer Biology, Department of Biochemistry and Molecular Biology, Fourth Military Medical University, Chang-Le Xi Street #127, Xi'an 710032, China; Cadet Team 6 of School of Basic Medicine, Fourth Military Medical University, Chang-Le Xi Street #127, Xi'an 710032, China; Department of Burns and Cutaneous Surgery, Xijing Hospital, Fourth Military Medical University, Chang-Le Xi Street #127, Xi'an 710032, China; The State Laboratory of Cancer Biology, Department of Biochemistry and Molecular Biology, Fourth Military Medical University, Chang-Le Xi Street #127, Xi'an 710032, China; Department of Prosthodontics, Beijing Stomatological Hospital and School of Stomatology, Capital Medical University, Tiantanxili Street #4, Dongcheng District, Beijing 100050, China; The State Laboratory of Cancer Biology, Department of Biochemistry and Molecular Biology, Fourth Military Medical University, Chang-Le Xi Street #127, Xi'an 710032, China; Department of Burns and Cutaneous Surgery, Xijing Hospital, Fourth Military Medical University, Chang-Le Xi Street #127, Xi'an 710032, China

**Keywords:** Diabetic foot, Foot ulcer, Engineered exosomes, Diabetic angiopathy, Diabetic peripheral neuropathy, Wound infection, Inflammation, Re-epithelialization, Wound healing

## Abstract

Diabetic foot ulcer (DFU), characterized by high recurrence rate, amputations and mortality, poses a significant challenge in diabetes management. The complex pathology involves dysregulated glucose homeostasis leading to systemic and local microenvironmental complications, including peripheral neuropathy, micro- and macro-angiopathy, recurrent infection, persistent inflammation and dysregulated re-epithelialization. Novel approaches to accelerate DFU healing are actively pursued, with a focus on utilizing exosomes. Exosomes are natural nanovesicles mediating cellular communication and containing diverse functional molecular cargos, including DNA, mRNA, microRNA (miRNA), lncRNA, proteins, lipids and metabolites. While some exosomes show promise in modulating cellular function and promoting ulcer healing, their efficacy is limited by low yield, impurities, low loading content and inadequate targeting. Engineering exosomes to enhance their curative activity represents a potentially more efficient approach for DFUs. This could facilitate focused repair and regeneration of nerves, blood vessels and soft tissue after ulcer development. This review provides an overview of DFU pathogenesis, strategies for exosome engineering and the targeted therapeutic application of engineered exosomes in addressing critical pathological changes associated with DFUs.

Highlights

- DFU pathologies of both pathogenesis at the systemic level and pathological abnormalities in the local wound microenvironment are reviewed.
- Engineering strategies of exosomes and their therapeutic application in crucial pathological changes of DFUs are summarized.
- Perspectives and current challenges of engineered exosomes-based therapy for DFUs are discussed.

## Background

Diabetes is one of the most lethal chronic diseases worldwide. In 2019, the global prevalence of diabetes was estimated to be 10.5% (536.6 million people), predicted to rise to 12.2% (783.2 million) by 2045 [1]. Among its severe complications is the diabetic foot ulcer (DFU), notorious for its high recurrence, amputation rates and mortality, with >40% of patients succumbing within 5 years [2]. DFU pathogenesis involves a combination of peripheral neuropathy and vascular insufficiency in diabetic patients, leading to ulcer formation and susceptibility to microbial invasion [3]. The intricate microenvironment of diabetic wounds, marked by hyperglycemia, ischemia, hypoxia, persistent infection and elevated reactive oxygen species (ROS), hampers the progression of ulcers beyond the inflammation phase of healing [4]. Current standard therapies include local pressure reduction, surgical debridement, revascularization, antibiotic therapy, wound dressing and glucose control; however, their efficacy remains limited [5–7]. Consequently, there is an urgent need to explore new methods for managing DFUs. To achieve improved clinical outcomes in DFU, it is important to tailor medications to the ulcer, or to combine and/or sequence multiple medications with integrated therapeutic properties [8, 9].

Advancements in nanotechnology offer promising prospects for precise drug selection and targeted delivery. Exosomes, nanoscale extracellular vesicles (EVs) originating from multivesicular bodies, emerge as key players in the diabetic wound microenvironment and serve as an alternative diagnostic and therapeutic platform to address limitations associated with traditional nanomedicines [10]. In contrast to artificially synthesized nanoparticles, exosomes possess natural origins and inherent biological properties that make them superior therapeutic carriers with a prolonged circulation half-life, low immunogenicity, biocompatibility, easy modifiability and payload stability [11, 12]. Notably, exosomes are enriched with bioactive components on their surfaces, including tetraspanins (such as CD9, CD63 and CD81) and adhesion molecules (like integrins and selectins), which enhance their modifiability and targeting capabilities [13]. Furthermore, the presence of specific receptors aids exosomes in evading the host immune system during circulation *in vivo*, exemplified by the ‘do not eat me’ signal molecule CD47 [14].

In both *in vivo* and *in vitro* studies, it has been demonstrated that exosomes from specific sources can modulate signaling pathways and cell function crucial in the pathogenesis and pathological abnormalities of DFUs. These exosomes enhance angiogenesis and peripheral nerve regeneration, combat microbial infections, suppress prolonged inflammation and stimulate wound re-epithelialization [15–17]. Developing engineered exosomes with enhanced curative properties, such as high yield, loading of the therapeutic compounds and precise targeting to specific sites, is essential for their utilization as regulated and practical drug delivery vehicles. This approach offers a safe, efficient and well-tolerated therapeutic strategy for treating DFUs [11, 18]. In this review, we outline the pathology of DFUs, discuss the engineering of exosomes and explore the therapeutic application of engineered exosomes in DFUs. Finally, we provide perspectives for future therapy and address current challenges. This review sheds light on the biological significance of exosomes and aids in the development of exosome-based nanotherapeutics for DFU.

## Review

### Pathology of DFUs

**Figure 1:**
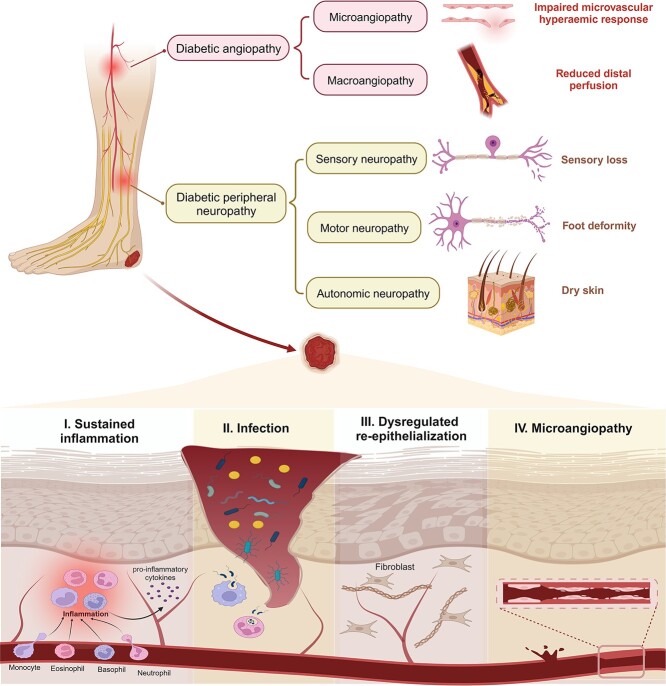
Pathology of diabetic foot ulcers. The primary predisposing factors for diabetic foot ulcers include diabetic angiopathy (leading to ischemic ulceration) and peripheral neuropathy (resulting in neuropathic ulceration). Moreover, pathological conditions within the wound microenvironment, such as microangiopathy, sustained inflammation, repeated infection and dysregulated re-epithelialization, contribute to the failure of ulcer healing. Figure created using BioRender (https://biorender.com/)

DFU arises from a complex interplay of various pathological changes linked to metabolic disorders in diabetes. Both systemic complications and local pathological events serve as significant risk factors for foot ulceration and contribute to the failure of ulcer healing (Figure 1) [19].

In the progression to ulceration, peripheral neuropathy and angiopathy [mainly observed in macrovascular events like peripheral artery disease (PAD)] are the primary causal factors that may independently or together contribute to neuropathic, ischemic or neuro-ischemic ulcers [19]. Abnormal glucose metabolism can directly cause nerve cell damage, Schwann cell apoptosis and demyelination, accompanied by impaired neurotrophic support, increased oxidative stress and inflammatory responses, which further exacerbate nerve injury [20, 21]. The combination of sensory and motor peripheral neuropathy results in reduced perception of temperature and pain stimuli, foot deformities and uneven biomechanical foot loading. This places individuals with advanced neuropathy at a heightened risk of falling and other unnoticed traumas [20, 22]. Autonomic neuropathy leads to reduced skin perfusion and dysfunctional sweat secretion, thereby impairing the skin’s barrier function [23]. Regarding diabetic macroangiopathy, PAD represents a manifestation of macrovascular events in the lower limbs, characterized by chronic arterial narrowing and occlusive disease due to atherosclerosis [24]. Chronic limb-threatening ischemia resulting from end-stage PAD is a significant aspect of the pathology and a defining feature of ischemic foot ulcerations [25]. This feature contributes to a poor prognosis for DFUs and increases the risk of adverse limb events and mortality [26, 27].

In preexisting foot ulcers, local pathological changes within the diabetic wound microenvironment, including microangiopathy, recurrent infections, dysregulated re-epithelialization and sustained inflammation, hinder the healing process. Abnormalities in nerve microvasculature result in reduced endoneurial perfusion and hypoxia. Consequently, impaired nerve axon reflex activities and microvascular regulation lead to irregular local blood flow [28]. This affects normal transport across capillary walls and diminishes the hyperemic response to injury, creating a pathological microvasculature environment. Moreover, impaired proliferation, migration and tube-formation capacity of human dermal microvascular endothelial cells in diabetic wounds also leads to inadequate angiogenesis [29]. The failure to meet tissue metabolic demands during foot skin stress or injury delays infection clearance and serves as a significant indicator of poor prognosis [30]. Persistent infection arises secondary to various complications, including neuropathy, immune cell dysfunction and immunological perturbations [31]. It remains the primary cause of hospitalization and amputation for diabetic patients [32, 33]. Delayed re-epithelialization also contributes to recurrent infections. Hyperglycemia and insulin resistance hinder rapid and effective re-epithelialization in diabetic wounds, impair skin barrier function and increase keratinocyte sensitivity to mechanical stress [34–38]. Moreover, stagnant inflammation obstructs signals for wound closure, perpetuating the cycle of persistent hypoxia, polymicrobial bioburden, and metabolic disorders of lipids and glucose [39]. Accumulative ROS, destructive enzymes and advanced glycation end products (AGEs) further prolong this inflammatory cycle [40, 41].

### Exosome preparation and engineering

**Figure 2:**
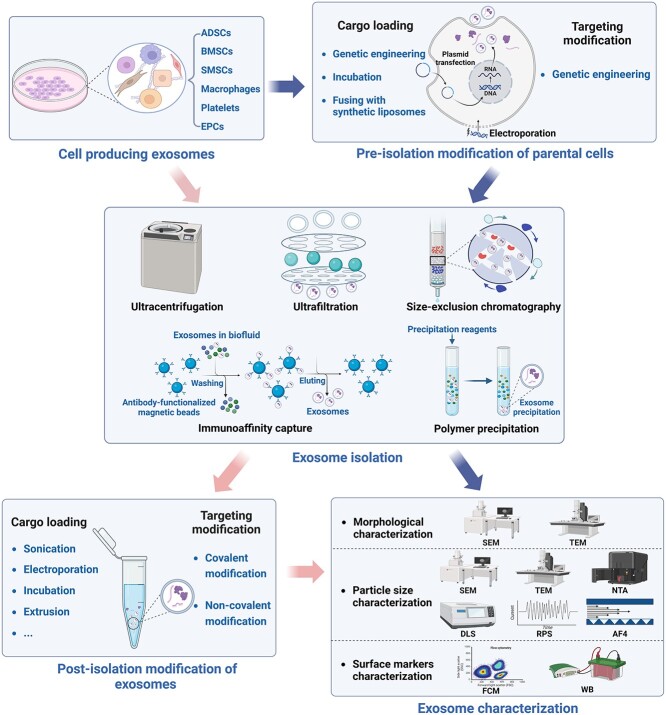
Schematic workflow of the two routes of engineered exosome preparation. Engineered exosomes produced through parental cell-based engineering undergo a sequential process involving pre-isolation modification of parental cells, followed by exosome isolation and characterization. If not modified before separation, exosomes can undergo post-isolation modification and subsequent characterization. In both routes, exosome engineering involves loading cargo and modifying targeting using various techniques. Figure created using BioRender (https://biorender.com/). *ADSCs* adipose-derived stem cells, *AF4* asymmetric flow field-flow fractionation, *BMSCs* bone marrow mesenchymal/stem stromal cells, *DLS* dynamic light scattering, *EPCs* endothelial progenitor cells, *FCM* flow cytometry, *NTA* nanoparticle tracking analysis, *RPS* resistive pulse sensing, *SEM* scanning electron microscope, *SMSCs* synovium mesenchymal stem cells, *TEM* transmission electron microscope, *WB* western blot

To enhance the effectiveness of exosomes at their intended site and improve the uptake of cargo molecules by cells, exosomes often need to encapsulate various therapeutic payloads and undergo surface modification through the attachment of targeting ligands [42]. To achieve this, novel bioengineering strategies have been actively investigated to enhance the stability, uniformity and safety of exosome-based delivery systems, aiming to improve their efficacy in treating DFUs and other diseases [12]. In this section, we discuss the current methods used to prepare engineered exosomes (Figure 2 and Table 1).

**Table 1:** Exosome engineering strategies to improve targeted delivery efficiency

**Category**	**Method**	**Mechanisms**	**Advantages**	**Disadvantages**	**Ref.**
Pre-isolation modification of parental cells					
Cargo loading	Incubation	Incubation of cargos with donor cells of exosomes and the subsequent isolation of cargo-carrying exosomes	Simple operation; no additional equipment required; suitable for small-molecule chemical drugs with low cytotoxicity	Low lading efficiency; drug cytotoxicity; impossible to control incorporation efficacy	[84–86]
	Genetic engineering	Expressing fusion proteins containing molecule-sorting modules (or the cargo itself) and exosome-localized proteins by genetic linkage	Enabling the recruitment of many molecules of interest into exosomes during exosome secretion; continuously replenishing required exosomes through the inherent exosome biogenesis process	Possibility of membrane protein alteration; low loading efficiency; gene expression change in donor cells; possibly toxic transfection agents	[70, 81, 82]
	Fusing with synthetic liposomes	Delivering functional agents to the parental cells via synthetic liposomes and subsequently producing cargo-carrying exosomes	Capable of loading various functional agents without modification of the native proteins and lipids	Low lading efficiency	[87, 88]
Targeting modification	Genetic engineering	Surface display of site-specific targeting peptides based on genetic linkage to exosomal membrane proteins	Preserving the native conformation and function of targeting proteins through the inherent protein expression mechanism and exosome biogenesis	Possibility of membrane protein alteration; a risk of endosomal degradation of peptides; gene expression change in donor cells; possibly toxic transfection agents	[65–68]
Post-isolation modification of exosomes					
Cargo loading	Sonication	Ultrasound treatment damaging the integrity of exosome membrane via mechanical shear force and allowing the entry of cargos during membrane deformation	High loading efficiency	Possible damage on exosomal membrane; the risks of exosome aggregation	[88–90]
	Electroporation	Small pores created in membrane under a short high-voltage pulse allowing for free diffusion of cargo into exosome	High loading efficiency; loading with large molecules and numerous drugs; ease in control	Destructive effects on protein structure; the risks of RNA aggregation; disrupting exosome integrity	[88–91]
	Incubation	Direct incubation of cargo with exosomes for a period of time	Simple operation; no additional equipment required	Low lading efficiency; drug cytotoxicity; impossible to control incorporation efficacy; drug hydrophobicity-dependent incubation efficiency	[86,88–90, 92]
	Extrusion	Loading of mixed exosomes and drugs into a lipid extruder with a porous membrane (aperture:100–400 nm)	High loading efficiency; uniform size of obtained exosomes	Possibly changing the properties of the secreted exosome membrane (e.g. zeta potential and membrane protein structure)	[89, 91, 93, 94]
	Freeze–thaw cycles	Incubating drugs with exosomes at room temperature, frozen rapidly at –80°C and thawed at room temperature, repeating three times	Simple operation; using mild conditions; rarely destroying bioactive substances; effective for various cargos	Possible exosome aggregation; unspecific loading efficiency; generally lower encapsulation rate than sonication or extrusion	[74, 95]
	Hypotonic dialysis	Dialyzing the membrane by stirring to load the drug exosomes	High loading efficiency of miRNAs and small interfering RNAs; higher efficiency than incubation	Broad peak of exosomal size distribution; damage on proteins and peptides in exosomes	[89, 95, 96]
	pH gradient	Internally acidified by a pH gradient method to promote the loading of negatively charged cargo	Maintaining the stability of nucleic acid drugs; comparable efficiency to sonication and electroporation	Reduction or aggregation of total protein in exosomes	[94]
Targeting modification	Covalent modification	Utilizing the mechanism of click chemistry (based on bio-orthogonal reaction) for the selective installation of targeting peptides on exosome surface	Attaching molecules on the surface of exosomes via alkyne-based cross-linking reactions without altering the size and characteristics of exosomes	Possibly toxic chemicals for inducing covalent bond; non-specific binding of targeting moiety over the exosome surface	[75–79]
	Non-covalent modification	Electrostatic interaction; receptor–ligand binding; hydrophobic insertion; aptamer-based surface modification	Less technical complexity	Possibility of membrane protein alteration; non-specific binding of targeting moiety over the exosome surface	[75, 76, 80]

### Biogenesis of exosomes

Exosomes, the smallest subtype of EVs compared to microvesicles and apoptotic bodies, typically have diameters ranging from 30 to 150 nm [10]. The biogenesis of exosomes occurs in three main stages: vesicle formation, cargo sorting and release of mature exosomes [43]. It begins with plasma membrane invagination, leading to the formation of early endosomes, followed by inward budding of the inner membrane, ultimately maturing into multivesicular bodies or lysosomes [44, 45]. These multivesicular bodies release intraluminal vesicles outside the cell upon fusion with the cellular plasma membrane, becoming exosomes [46]. Due to differences in their biogenesis patterns, EV subtypes vary in physical properties such as, morphology, particle size, structure and density, and cargo recruitment [47, 48]. The cellular compositions within different EV subtypes are not randomly assigned but consist of specific subsets of integrated RNA, proteins and lipids through active sorting mechanisms [49, 50]. Recent research has highlighted differences in RNA profiles between exosomes and microvesicles, indicating a higher consistency in RNA expression within exosomes, suggesting their potential as a stable and repeatable natural nano-delivery system for therapeutic applications [51]. Exosomes can be distinguished from other subtypes based on particle size and specific surface markers such as CD63, CD81 and CD9 [52]. Various cell types serve as sources of exosomes, including adipose-derived stem cells (ADSCs), bone marrow mesenchymal stem cells (BMSCs), synovium mesenchymal stem cells (SMSCs), macrophages, platelets and endothelial progenitor cells (EPCs). The functionality of exosomes is closely linked to the type and physiological state of their parental cells, underscoring the importance of selecting the appropriate cellular source.

### Isolation methods for exosomes

Efficient and pure isolation methods for exosomes are crucial for advancing both basic research and clinical translation of exosomes. Various isolation methods have been employed to separate exosomes from different fluids such as plasma, urine and milk. Among these, ultracentrifugation, including both differential and density gradient ultracentrifugation, is the most commonly used method in basic research [53]. This technique segregates exosomes from other EVs and the extracellular matrix (ECM) based on differences in their sedimentation coefficients [54]. Although ultracentrifugation remains the most widely used technique for exosome isolation and offers advantages in terms of purity [55, 56], its downstream analysis is limited due to factors such as time consumption, operator sensitivity, structural damage and coprecipitation with soluble proteins [57–59]. Other isolation techniques, based on physical characteristics such as shape, size and density, include ultrafiltration, size-exclusion chromatography and polymer precipitation. Compared to ultracentrifugation-based exosome isolation, the first two methods based on molecular size offer significant time and labor savings and do not require a specific ultracentrifuge machine [60], although they may compromise purity, cost and yield [61, 62]. Precipitation, the basis for many commercial exosome separation kits, is popular in basic exosomal research due to its low cost and simplicity. However, it primarily serves as a concentration and enrichment tool rather than a true exosome isolation method because the precipitation reagents (mostly polyethylene glycol) are not fully removed from the final product [59, 63]. The immunoaffinity technique isolates exosomes by utilizing the interaction between exosomal surface proteins and specific antibodies/aptamers immobilized on magnetic beads or other matrices [64]. While this technique offers high specificity and precision for exosome isolation, the dissociation of captured exosomes remains a significant challenge, and their biological activity can be affected by the eluent.

### Engineering strategies for exosomes

#### Targeting modification of exosomes

Parental cells have the ability to produce exosomes attached to site-specific targeting peptides through genetic linkage to certain fusion partners [65–68]. These partners include exosomal membrane-localized proteins such as lactadherin, lysosome-associated membrane protein-2b (Lamp2b), glycosyl-phosphatidyl-inositol, and tetraspanins such as CD63, CD9 and CD81 [69]. For example, modified exosomes fused with rabies virus glycoprotein attached to Lamp2b have shown efficient delivery of therapeutic molecules into the brain [65, 66]. Moreover, researchers are exploring additional exosomal membrane proteins for surface display. PTGFRN, a scaffold protein abundant in EVs derived from various cell types, have been identified as capable of effectively incorporating targeting ligands onto the EV surface at therapeutically relevant levels by fusing to the exposed N terminus [70]. Its unique topology makes it an attractive scaffold for surface display compared to tetraspanins, where both N and C termini are sequestered in the lumen [71, 72]. This exploration of novel membrane scaffold proteins expands the practical approach to customizing exosomes for targeted delivery to specific cells. By circumventing the limitations of post-isolation surface modification, parental cell-based engineering preserves the native conformation and function of targeting proteins through the inherent protein expression mechanism and exosome biogenesis [67]. However, there is a risk of endosomal degradation of peptides, which could affect the yield of peptide-functionalized exosomes [68]. Additionally, targeting ligands can be transferred onto the membranes of exosome-producing cells through fusion with liposomes. Exosome–liposome hybrids enhance target cell uptake of exosomes due to improved pharmacokinetic profiles and enhanced colloidal stability [73, 74].

Covalent modification of exosomes commonly involves click chemistry, a mechanism where copper-catalyzed azide–alkyne cycloaddition-based reactions are the most versatile bio-orthogonal reactions [75–79]. This method allows for the selective installation of targeting peptides onto the surface of exosomes [77]. With the advancement of copper-free click chemistry and its combination with metabolic glycoengineering, click chemistry has become less cytotoxic and more widely applicable in exosome-based targeted delivery [78, 79]. Another type of direct exosome engineering involves noncovalent conjugations through electrostatic interactions, receptor–ligand binding, hydrophobic insertion and aptamer-based surface modification [75, 76, 80]. These approaches offer the advantage of less technical complexity and are extensively used to attach targeting moieties to the membrane of exosomes [75, 80].

#### Drug loading into exosomes

Attaching exogenous cargo to the surface of exosomes or packaging it inside the lumen is a crucial step in creating therapeutic nanocarriers. Through genetic engineering, cells producing exosomes can express fusion proteins containing molecule sorting modules (or the cargo itself) and exosome-localized proteins [70, 81, 82]. These modules enable the recruitment of many molecules of interest into exosomes during exosome secretion, and the required exosomes can be continuously replenished through the inherent exosome biogenesis process [70, 82]. For instance, Sutaria *et al*. devised a system in HEK293T cells to generate exosomes with enhanced miR-199a loading. They fused modified pre-miR-199a to the transactivation response RNA element and separately fused the transactivation of transcription protein to Lamp2a [83]. By introducing the transactivation response RNA/ transactivation of transcription peptide interaction between the exosomal surface protein Lamp2a and pre-miR-199a, the miRNA cargo was effectively encapsulated into the lumen of exosomes, resulting in a 65-fold increase in loading. This same strategy holds promise for efficiently loading any therapeutic miRNA into exosomes. Additionally, common approaches to producing exosomes loaded with therapeutic agents (such as therapeutic RNAs, small molecule drugs, proteins and peptides) include incubating producer cells with cargos or fusing producer cells with synthetic liposomes [84–88].

Direct exosome engineering has been widely utilized for loading small nucleic acid molecules (e.g. small interfering RNAs and miRNAs) and exogenous therapeutic drugs into exosomes. Methods such as sonication [88–90], electroporation [88–91], incubation [86,88–90, 92], extrusion [89, 91, 93, 94], freeze–thaw cycles [74, 95], hypotonic dialysis [89, 95, 96] and pH gradient [94] have been employed for this purpose. These post-isolation engineering methods offer an advantage over producer cell-based loading in terms of reduced technical complexity. However, they require repetitive operations because they lack a continuous source of exosomes [81].

### Characterization of exosomes

Exosomes can be characterized based on their morphology, size and protein composition. Commonly used techniques for detecting exosome morphology and size include transmission electron microscopy, scanning electron microscopy, and transmission scanning electron microscopy, which is an application of scanning electron microscopy in transmission mode [97]. In addition to direct observation by electron microscopy, other methods such as nanoparticle tracking analysis, resistive pulse sensing, dynamic light scattering and asymmetric flow field-flow fractionation can be employed to determine exosome size distribution [98, 99]. Exosomes can also be identified based on surface protein markers using western blotting and flow cytometry. These markers include tetraspanins (CD9, CD63 and CD81), integrins, Hrs (an ESCRT-0 protein), tumor susceptibility gene 101 and Alix, which are commonly detected [100].

### Therapeutic effect of engineered exosomes in DFUs

**Figure 3:**
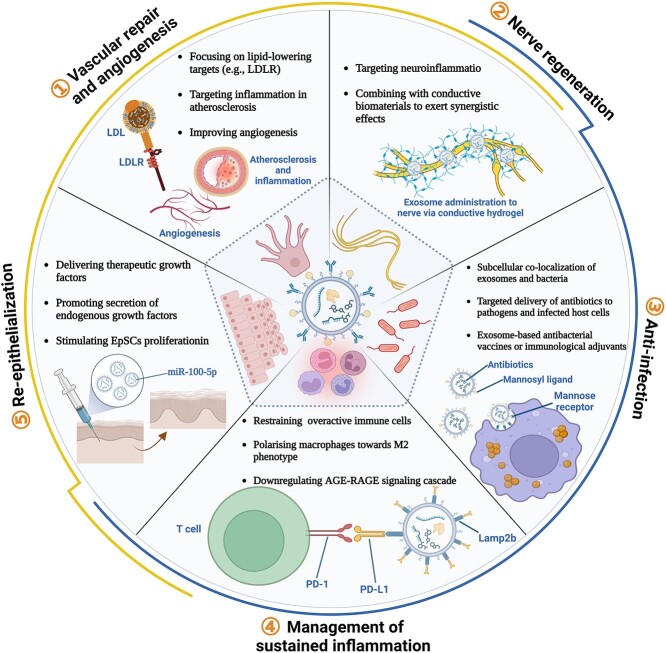
The role of engineered exosomes in the treatment of diabetic foot ulcers. The application of engineered exosomes targets the pathogenesis and pathological microenvironment of diabetic foot ulcers, including vascular repair and angiogenesis, nerve regeneration, anti-infection measures, management of sustained inflammation, and re-epithelialization. Figure created using BioRender (https://biorender.com/). *LDL* low-density lipoprotein, *LDLR* low-density lipoprotein receptor, *Lamp2b* lysosome associated membrane protein-2b, *EpSC* epidermal stem cells, *AGE* advanced glycation end products, *RAGE* receptor for AGE

Exosomes derived from specific sources (e.g. ADSCs, BMSCs, SMSCs, macrophages, platelets and EPCs) play a role in regulating specific signaling pathways and cell functions within the crucial pathogenesis and pathological microenvironment of DFUs. In this section, we concentrate on the utilization of engineered exosomes in experimental and clinical therapeutics for DFUs (Figure 3 and Table 2).

**Table 2:** Therapeutic role of engineered exosomes in the pathologies of diabetic foot ulcers

**Category**	**Origin of exosomes**	**Experimental models**		**Surface modified ligands**	**Cargos**	**Delivery methods**	**Therapeutic outcomes**	**Ref.**
		***In vivo***	***In vitro***					
Vascular repair and angiogenesis	AML12 cells	Ldlr−/− mice fed a high-fat diet	HEK 293 T cells with low endogenous LDLR expression and AML12 hepatocytes	–	Ldlr mRNA	Tail vein injection	Decreased lipid deposition in the liver, lowered the serum LDL-cholesterol level, and reduced atherosclerotic plaques and inflammation	[109]
	M2 macrophage	Acute peritonitis mouse model and early atherosclerosis mouse model	Activated HUVECs， common inflammatory cells and typical foam cells	Chemokine receptors	HAL, anti-inflammatory cytokines	Intravenous injection	Promoted intrinsic biosynthesis and metabolism of heme to generate anti-inflammatory carbon monoxide and bilirubin, and alleviated atherosclerosis	[119]
	Bone marrow-derived macrophages	Apoe −/− mice fed a Western diet	Bone marrow-derived macrophages	–	miRNA-99a/146b/378a	Intraperitoneal injection	Modulated M2 polarization, reduced myeloid cells in the circulation and macrophages in aortic root lesions, decreased necrotic lesion areas in atheromas	[121]
	ADSCs	STZ-induced diabetic rats	EPCs	–	Nuclear factor-E2-related factor 2	Not reported	Promoted proliferation and angiopoiesis in EPCs, increased granulation tissue formation and angiogenesis, reduced levels of inflammation and ulcerated area	[130]
	ADSCs	Sprague–Dawley rats with full-thickness skin defects	Fibroblasts, HUVECs	–	miR-126-3p	Topical injection	Promoted the proliferation and migration of fibroblasts and angiogenesis of HUVECs, increased collagen deposition, promoted wound healing of full-thickness skin defects	[136]
	SMSCs	STZ-induced diabetic rats	Human fibroblasts, HMEC-1 cells	–	mir −126-3p	Chitosan hydrogel	Stimulated the proliferation of HDFs and HMEC-1, promoted migration and tube formation of HMEC-1, accelerated re-epithelialization, activated angiogenesis, and promoted collagen maturity	[137]
Nerve re-generation	MSCs	BKS.Cg-m+/+Leprdb/J (db/db) mice	Macrophages, human dermal microvascular endothelial cells	–	miR-146a	Tail vein injection	Increased nerve conduction velocity, lowered thermal and mechanical stimuli threshold, inhibited the activation of peripheral blood inflammatory monocytes and endothelial cells	[152]
	BMSCs	STZ-induced diabetic rats	Neural cell lines, SH-SY5Y and Neuro2a cells	–	Polypyrrole nanoparticles	Intramuscular injection	Normalized the nerve conduction velocity and compound muscle action potential, restored gastrocnemius muscle morphology, muscle mass and integrity	[155]
	BMSCs	STZ-induced diabetic rats	PC-12 cells, Schwann cells and RAW264.7 cells	–	–	Electroconductive hydrogels	Promoted the attachment and migration of Schwann cells, inhibited inflammation by modulating M2 macrophage polarization, facilitated myelinated axonal regeneration	[159]
	Adipose-derived stem cells	Sciatic nerve injury model	PC-12 Cells	–	miR-218	Tail vein injection	Facilitated the regeneration of injured sciatic nerves, motor and nerve fibers, combined with engineered nanofibrous scaffold	[160]
Anti-infection	Mouse RAW264.7 macrophages	MRSA peritonitis model	MRSA-infected macrophages	–	Linezolid	Subcutaneous injection	Overcome intracellular infections by MRSA	[173]
	Mouse RAW264.7 macrophages	MRSA-infected mice model	MRSA-infected macrophages and A549 cells	Mannosyl ligands	Lysostaphin and vancomycin	Intravenous injection	Overcome intracellular infections by MRSA	[79]
Anti-inflammation	SK-MEL-5 cells, B16F10 cells	Balb/c mice with full-thickness skin wound	Peripheral blood mononuclear cells, HaCaT cells, HDFs	PD-L1	–	PF-127 thermoresponsive hydrogel	Decreased T cell proliferation, reduced CD8+ T lymphocytes in the spleen and lymph nodes during inflammation phase, speeded up skin cell migration *in vitro* and wound healing *in vivo*	[202]
	EPCs	STZ-induced diabetic rats	–	–	miRNA-221-3p	Topical application	Targeted AGE–RAGE, cell cycle and p53 signaling pathway, promoted skin wound healing in diabetic mice	[220]
Re-epithelialization	Platelets	New Zealand white rabbits (*Oryctolagus cuniculus*) with full-thickness skin defects	HUVECs, primary rabbit dermal fibroblasts and human keratinocyte	–	TGF-β	Surgical fibrin sealant	Promoted full-thickness wound healing with the regrowth of hair follicles and sebaceous glands, enhanced angiogenesis, and facilitated collagen synthesis and restoration of dermal architecture	[232]
	Platelet-rich plasma	STZ-induced diabetic rats	HMEC-1, primary dermal fibroblasts	–	Principal growth factors from platelets	Sodium alginate hydrogel	Promoted proliferation and migration of endothelial cells and fibroblasts, enhanced angiogenesis and re-epithelialization in diabetic wounds	[233]
	MSCs	STZ-induced diabetic rats	High glucose co-culture system of HaCaT cells and HDFs	–	miR-155 inhibitor	Subcutaneous injection	Promoted keratinocyte migration, restoration of FGF-7 levels and anti-inflammatory action, and enhanced collagen deposition, angiogenesis and re-epithelialization	[238]
	ADSCs	–	EpSCs, human skin tissue explants	–	miR-100-5p	Intradermal injection	Promoted EpSCs proliferation through myotubularin-related protein 3-mediated elevation of phosphatidylinositol-3,4,5-trisphophate and activation of AKT and ERK, enhanced skin wound healing	[247]
	EpSCs	Db/db mice	Primary diabetic fibroblasts and macrophages	–	–	Topical injection	Promoted the proliferation and migration of diabetic fibroblasts and macrophages, induced M2 macrophage polarization, enhanced angiogenesis, and accelerated diabetic wound healing	[248]

*ADSCs* Adipose-derived stem cells, *AML12 cells* alpha mouse liver cells, *BMSCs* bone marrow mesenchymal/stem stromal cells, *EPCs* endothelial progenitor cells, *EpSCs* epidermal stem cells, *FGF* fibroblast growth factor, *HaCaT cells* human clonal keratinocytes, *HAL* hexyl 5-aminolevulinate hydrochloride, *HDFs* human dermal fibroblasts, *HUVECs* human umbilical vein endothelial cells, *LDLR* low-density lipoprotein receptor, *miRNA/miR* microRNA, *MRSA* methicillin-resistant *Staphylococcus aureus*, *MSCs* mesenchymal stromal cells, *RAGE* receptor for AGEs, *SMSCs* synovium mesenchymal stem cells, *STZ* streptozotocin, *TGF-β* transforming growth factor beta

### Vascular repair and angiogenesis

Diabetic angiopathy, classified as macroangiopathy or microangiopathy, is the primary predisposing factor for ischemic ulceration in the lower limbs, contributing significantly to disease burden [101, 102]. PAD, characterized by atherosclerotic events in the lower extremity macrovessels, requires prompt intervention to prevent and treat ischemic DFU effectively [25, 27]. Additionally, enhancing wound vascularization to improve tissue blood supply and alleviate hypoxia is a critical aspect of DFU treatment.

In the diabetic population, metabolic abnormalities such as oxidative stress, chronic inflammation, and the accumulation of AGEs and low-density lipoprotein cholesterol (LDL-C) complicate the pathophysiological mechanisms of PAD more than in non-diabetic individuals [103]. Although antihyperglycemic agents and lipid-lowering therapies have shown clinical utility in reducing atherosclerosis-related macrovascular events, the risk of PAD remains high in diabetes [104–106]. Hence, there is an urgent clinical need for novel lipid-lowering agents that target inflammatory pathways, LDL-C and other atherogenic particles [107]. The LDL receptor (LDLR) plays a crucial role in the endocytosis of LDL particles and the removal of excess LDL-C from serum [108]. Increasing the density of normally functioning LDLR is an effective solution to elevated LDL-C levels in plasma. In a study by Li *et al*., exosomes encapsulating therapeutic LDLR mRNA were generated from donor alpha mouse liver cells (AML12 cells) with forced expression of LDLR [109]. They demonstrated that exosome-mediated LDLR mRNA in mouse models could robustly restore LDLR expression, minimize and stabilize atherosclerotic plaques, and reverse phenotypes such as steatosis, high LDL-C and atherosclerosis. LDL remains a primary treatment target in clinical practice. Additionally, several new targets, including lipoprotein(a) [110], apolipoprotein C-III [111], angiopoietin-like proteins 3 and 4 [111, 112], ATP citrate lyase [113], and triglyceride-rich lipoproteins and their remnants [114], have emerged as therapeutic candidates in lipid management and the treatment of atherosclerotic macroangiopathy. Nucleic acid-based approaches to target these newly recognized mediators have made significant progress [115], and engineered exosomes serve as compelling nanocarriers of nucleic acid agents [11].

In addition to focusing on lipid-lowering drugs, persistent inflammatory responses aggravate the progression of atherosclerosis and can eventually lead to plaque disruption [116]. Therefore, insufficient reduction in inflammation during lipid-lowering therapy may contribute to treatment failure and recurrent atherosclerotic events. A study involving patients with stable atherosclerosis found that systemic treatment with a low-dose anti-inflammatory drug methotrexate did not decrease levels of plasma inflammatory factors or the occurrence of atherosclerotic disease compared to placebo [117]. This suggests that targeted delivery of anti-inflammatory drugs to activated proinflammatory cells using nanocarriers may be preferable to systemic delivery for resolving inflammation and minimizing adverse effects [118]. Several trials have targeted pivotal inflammatory signaling networks to pursue valuable therapies. Promising strategies include exosome-based targeted administration of anti-inflammatory miRNAs, small molecule drugs, and inhibitors that target the NACHT, LRR, and PYD domains-containing protein 3 (NLRP3) inflammasome or other signaling pathways promoting the release of proinflammatory cytokines [119–121]. Moreover, different binding peptides targeting subendothelial collagen IV [122], vascular cell adhesion molecule-1 [123], endothelial cell integrins [124] and several epitopes overexpressed on infiltrated macrophages and foam cells [125–127] can be attached to the surface of exosomes. This decoration enhances exosome accumulation in atherosclerosis lesions and internalization by lesional cells. For instance, exosomes derived from M2-like macrophages, engineered to exhibit inflammation-tropism and intrinsic biosynthesis functionality, were used to deliver hexyl 5-aminolevulinate hydrochloride, an US Food and Drug Administration (FDA)-approved drug that initiates the biosynthesis and metabolism of heme and stimulates the production of anti-inflammatory compounds such as carbon monoxide and bilirubin [119]. After systemic administration, these engineered exosomes targeted inflammatory endothelial cells and accumulated in atherosclerotic lesions due to various chemokine receptors displayed on their surface. This targeting mediated anti-inflammatory and anti-atherosclerotic effects through the release of endogenous anti-inflammatory cytokines and exogenous hexyl 5-aminolevulinate hydrochloride [119].

In addition to macroangiopathy, diabetic foot disease involves the loss of microvessels and slow blood vessel reconstruction, leading to delayed wound healing [3, 128]. EPCs play a crucial role in angiogenesis by promoting the growth of new blood vessels through migration, proliferation, adhesion, differentiation into endothelial cells and secretion of angiogenic growth factors that are necessary for mobilizing tissue-residing progenitor cells [129]. Therefore, enhancing EPC-dependent angiogenesis or using small molecules transferred intercellularly by EPC-exosomes (EPC-Exos) to stimulate angiogenic and regenerative responses of recipient cells represent promising targets for exosome-based therapy [130, 131]. Exosomes derived from ADSCs overexpressing nuclear factor-E2-related factor 2 have been found to prevent the glucose-induced senescence of EPCs and enhance wound vascularization and healing, potentially by reducing ROS and inflammatory cytokine expression [130]. miR-126, an endothelial-specific miRNA, plays a crucial role in EPC proliferation, differentiation and migration [132]. In response to vascular injury and/or hypoxia, miR-126 is upregulated in EPCs and endothelial cells, promoting neovessel formation and vascular repair by mediating the proangiogenic signal transduction pathways involving fibroblast growth factor and vascular endothelial growth factor [133, 134]. Studies have shown that miR-126 is downregulated in EPCs derived from diabetic patients, leading to reduced colony formation, proliferation and migration, and reduced apoptosis of EPCs by targeting SPRED-1 and phosphoinositol-3 kinase regulatory subunit 2 [133, 135]. Ma *et al*. demonstrated the effects of exosomes derived from ADSCs transfected with miR-126-3p mimics in promoting the proliferation and migration of fibroblasts and angiogenesis of human umbilical vein endothelial cells in a normal rat full-thickness skin wound model [136]. In a diabetic rat model of full-thickness skin defects, exosomes derived from synovium mesenchymal stem cells loaded with miR-126-3p combined with chitosan exhibited proangiogenic and re-epithelialized effects [137].

### Nerve regeneration

Diabetic peripheral neuropathy (DPN) is a prevalent and severe complication of diabetes mellitus, affecting at least half of diabetic patients over time [138, 139]. It typically manifests as symmetrical and length-dependent neuropathy [140], primarily affecting long nerves like the sural nerve, initially involving the distal feet [141]. However, pharmacotherapies targeting the underlying pathogenesis, such as α-lipoic and epalrestat, face limitations due to their highly hydrophobic nature, low bioavailability and poor pharmacokinetic properties [142, 143]. Therefore, designing novel agents to address the pathogenetic mechanisms of DPN is crucial to facilitate peripheral nerve regeneration, treat neuropathic ulcers, restore muscle strength in distal limbs and improve perception of external stimuli and skin barrier function. Engineered exosomes offer an ideal platform for these purposes.

Low-grade inflammation has been shown to play a role in the development of DPN, highlighting the potential effectiveness of targeting neuroinflammation as a mechanism-based strategy for DPN treatment [144, 145]. Exosomes serve as versatile carriers for both endogenous and exogenous anti-inflammatory molecules, offering tissue-specific targeting and reduced side effects. While mesenchymal stromal cells (MSCs) have demonstrated neuroprotective effects by promoting the secretion of neurotropic factors and aiding nerve repair in DPN [146], their clinical use is limited due to the long induction period and potential risk of tumor formation [147]. MSC-Exos, as major paracrine effectors of MSCs, contain numerous neuroprotective and anti-inflammatory small molecules and proteins [148, 149], presenting a promising alternative therapy for DPN [150]. Specific miRNAs inherited from parent cells, such as let-7a, miR-23a and miR-125b, contribute to reliving neuroinflammation by targeting the Toll-like receptor 4/nuclear factor-κB signaling pathway [151]. Modifying exosomes to carry additional functional miRNAs can enhance the therapeutic efficacy of MSC-Exos in peripheral nerve repair in diabetes by overcoming the variability in miRNA content and functionality dependent on the parental cell condition [150]. Fan *et al*. proved that treatment of DPN with MSC-Exos enriched with miR-146a significantly increased intraepidermal nerve fiber density, nerve conduction velocity, and sensitivity to thermal and mechanical stimuli in diabetic mice [152]. Additionally, miR-17-92-enriched MSC-Exos have demonstrated greater effectiveness in promoting axonal growth by activating the phosphatase and tensin homolog (PTEN)/mammalian target of rapamycin (mTOR) signaling pathway in recipient neurons compared to native exosomes [153].

Recent research has focused on the synergistic effect of exosomes and biomaterials in nerve regeneration following DPN, aiming to develop novel tissue-engineered nerve grafts. Electrically conducting polymers enable targeted delivery of current to injured nerves, enhancing the precision of electrical stimulation treatment [154]. For instance, Singh *et al*. developed an axosomal system by fusing BMSCs-Exos and liposomes containing conducting polypyrrole nanoparticles. This system, combined with electrical stimulation, provided both biochemical and electrical guidance cues for axonal myelination and regeneration after DPN [155]. Conductive hydrogel, another electric conductor, mimics the biological and electrical characteristics of human endogenous nerve tissue and serves as an ideal slow-release carrier for exosomes [156]. It facilitates myelinated axonal regeneration, thus restoring muscular locomotor function after denervation atrophy [157, 158]. In a study by Yang *et al*., electroconductive hydrogel loaded with BMSCs-Exos was used to treat DPN, promoting axonal regeneration and remyelination through the MEK/ERK pathway, and offering long-acting relief of inflammatory pain by enhancing M2-polarized macrophages via the nuclear factor-κB pathway [159]. Furthermore, a combination of miR-218-enriched exosomes and an engineered scaffold was found to promote the recovery of damaged sciatic nerves, suggesting a potential clinical application in improving motor function in patients with peripheral nerve injury [160]. These tissue-engineered nerve grafts combine biomimetic qualities, exosome-based cargo delivery and electrical conductivity to function synergistically with electrical stimulation. They hold promise for alleviating sensory loss and muscle atrophy in patients’ distal lower extremities, preventing ulceration and promoting the recovery of existing plantar neuropathic ulcers.

### Anti-infection

The regular occurrence of infection poses a challenge to managing DFUs [32]. While oral or intravenous antibiotics are currently standard treatment for diabetic foot infections, their systemic administration can lead to potential adverse effects such as ototoxicity and nephrotoxicity. Additionally, microcirculation disorders can result in inadequate local drug concentrations [161, 162]. Therefore, there is a pressing need to develop local antimicrobial treatments to minimize systemic exposure and improve drug accumulation and penetration in biofilms [163]. Tailor-made exosomal systems show significant potential in optimizing antibiotic dosing and developing antibiotic-independent antimicrobial agents.

An exosome-based delivery system offers a promising approach to combat intracellular infections by enhancing the penetration and accumulation of antibiotics within infected host cells. This is crucial for reducing recurrent infections and mortality in susceptible DFU patients. Among the important pathogens associated with diabetic foot infections [32], *Staphylococcus aureus* [164, 165], *Pseudomonas aeruginosa* [166], *Acinetobacter baumannii* [167], *Klebsiella pneumoniae* [168] and especially Methicillin-resistant *S. aureus* (MRSA) [169] are known to invade and survive within eukaryotic host cells. MRSA is one of the most prevalent multidrug-resistant pathogens, occurring in ~16.78% of diabetic foot infections [170]. MRSA and other pathogens have the ability to invade and survive within eukaryotic host cells, allowing them to evade antibiotics and host immune responses, leading to treatment failure [171–173]. Recent studies have demonstrated that exosomes loaded with linezolid can be effectively taken up by macrophages infected with MRSA due to the phagocytic nature of these cells. This results in enhanced accumulation of exosome-encapsulated antibiotics within macrophages, leading to more pronounced bactericidal effects on intracellular MRSA compared to free linezolid [173]. Moreover, linezolid-loaded exosomes exhibited strong co-localization with lysosomes. Since a significant fraction of the total intracellular MRSA is present in lysosomes as well, the colocalization of exosomes with bacteria in the same intracellular compartments could improve bactericidal efficiency relying on high local concentrations of antibiotics delivered by exosomes [174, 175]. To further improve cell uptake and decrease toxicity and resistance, exosomes can be decorated with targeting ligands against infected host cells [79]. For instance, mannose-conjugated exosomes loaded with lysostaphin and vancomycin have been developed for targeted antibiotic delivery to eliminate both quiescent and metabolically active MRSA in macrophages [79]. Mannose receptors, such as CD206, which mediates macrophage targeting, can be utilized [176]. Additionally, other well-characterized membrane molecules such as phosphatidylserine [177] and anti-macrophage receptor with collagenous structure (MARCO) antibody [178, 179] could be explored to modify the surface of exosomes for recognition by phagocytes and for treatment of intracellular infection.

Moreover, in the ulcer microenvironment where pathogenic bacteria colonize, exosomes can be modified with bacterium-specific ligands to actively target and enhance the localization of antibiotics to pathogens. Various ligands, such as lysostaphin [180], vancomycin [181–183], single-domain antibody [184], bacteriophage tailspike proteins [185] and aptamer [186, 187], have been extensively studied and conjugated to nanoparticles, presenting potential applications for exosomal surface modifications. Despite the use of numerous nanoparticles for clinical antibiotic delivery, complex targeting strategies often fail to optimize synthetic nanosystems [188]. The process of incorporating ligands or domains onto nanoparticle surfaces involves multiple ‘top-down’ chemical conjugation steps and labor-intensive work, often utilizing organic solvents and highly reactive chemical reagents that are environmentally unfriendly [189]. Recently, biomimetic nanoparticles derived from exosome membranes have emerged as a promising alternative for targeted drug delivery, offering additional functionality such as immune evasion and prolonged blood circulation time [190]. Various techniques, including incubation, extrusion, sonication, freeze–thaw methods and microfluidic sonication, are used to coat nanoparticles with exosome membranes to form core–shell nanostructures or to fuse exosomes with lipid nanoparticles to form hybrid particles [190, 191]. These processes involve independent processing of exosome membranes and particle cores before coating or fusion. Although biomimetic nanoparticles/nanovesicles have been successfully utilized for immune evasion-mediated targeting delivery to homologous tumors [190], there have been no reports on their application in enhancing binding and targeted antimicrobial drug delivery to pathogens. Exploring this alternative platform could provide a direction for effective local treatment of infected DFUs.

Engineering exosomes to selectively target or synergistically engage the innate and adaptive immune systems offer a promising approach to enhance immune responses against infection [192]. In naturally occurring infection, exosomes play a potential role in presenting pathogens to activate host defense and immune responses, leading to the release of inflammatory factors and enhancing resistance against microbial invasion [193]. Consequently, exosomes are emerging as effective immune response activators during the infection process and have shown promise in the development of antibacterial vaccines or immunological adjuvants. While engineered exosomes have shown significant progress as therapeutic cancer vaccines, there is limited research on their application in alleviating bacterial wound infections [194]. Exploring this avenue further holds promise for future studies.

### Management of sustained inflammation

In acute wound healing, the initial local inflammatory responses in the wound bed serve as an immunological barrier against microbes and are soon followed by the proliferative phase [195]. However, chronic ulcers in diabetic foot disease undergo a prolonged and intensified inflammatory phase, disrupting orderly healing and leading to intractable ulcers that are challenging to manage long-term [196]. The inflammatory wound environment is characterized by an abundance of neutrophils, sustained release of proinflammatory cytokines (such as tumor necrosis factor-α and interleukin-1β), a high presence of macrophages with a dysfunctional phenotype, and dysregulation of immunoregulatory molecules like adenosine and nitric oxide [197–199]. Therefore, strategies aimed at regulating overactive immune cells and dysregulated inflammatory signaling offer a promising therapeutic option for nonhealing diabetic wounds [200]. Consistent with this idea, a recent study revealed that programmed death-ligand 1 (PD-L1) exhibits anti-inflammatory and proliferative activities in healing defects by activating the eIF3I–PD-L1–IRS4 axis, suggesting its potential as a regulator involved in re-epithelialization and the inflammatory response in DFUs [201]. In another study by Su *et al*., exosomes expressing high levels of PD-L1 were found to bind specifically to PD-1 on T cells, inhibiting their proliferation and cytokine production [202]. Additionally, exosomal PD-L1 was shown to promote the migration of both human clonal keratinocytes (HaCaT cells) and human dermal fibroblasts *in vitro* and accelerate wound contraction and re-epithelialization *in vivo*.

Moreover, the impaired transition of macrophages from the proinflammatory M1 to the anti-inflammatory M2 phenotypes in DFUs is closely associated with a deficient switch from inflammation to repair, reduced angiogenesis and diminished collagen deposition [199, 203]. Research indicates that 13 proinflammatory cytokines are upregulated in hyperglycemic environments [204], leading to increased polarization of M1 macrophages. Additionally, sustained elevated glucose levels enhance the metabolic activity of M1 macrophages and amplify their responses to cytokine stimulation [204–206]. Recent studies have shown that gene regulation through epigenetic modifications and miRNA can influence the dynamic plasticity of macrophages. Targeting these modifications or their transcripts holds promise for enhancing the anti-inflammatory properties of macrophages and their wound repair capacity [207, 208]. Li *et al*. developed an engineered exosome-based strategy to reduce the abundance of RNA targets by utilizing HuR (an RNA-binding protein) bound to Lamp2b (an exosomal membrane-localized proteins) to mediate the specific RNA degradation in lysosomes, that is specifically effective in macrophages [209]. This system offers a therapeutic approach to intervene in inflammation-related RNA function and alleviate macrophage-mediated inflammatory responses in chronic wounds. Furthermore, strategies focusing on targeting macrophage polarization to adapt their phenotype to the wound microenvironment have been widely studied, mainly aiming to reverse M1 macrophages into the anti-inflammatory M2 phenotype [210, 211]. However, besides the consideration of impaired macrophage polarization on chronic wound healing, the timing of phenotypic switching is crucial. Although, the presence of substantial M2 macrophages is associated with improved healing, M1 macrophages are essential for initiating early inflammation and advancing diabetic wound healing into the next phase [203,212–214]. Therefore, exosomal-based therapeutics targeting macrophage polarization should be engineered based on the inflammatory progression of diabetic wound healing, aiming to overcome the persistent inflammatory phase in DFUs [212].

Additionally, AGEs, which constitute a diverse array of chemical compounds, are generated and accumulated throughout the body as a result of extensive nonenzymatic glycation reactions in diabetes. AGEs remain significant risk factors for various diabetic complications [215]. The excessive activation of the receptor for AGEs (RAGE) due to increased AGE levels in chronic wounds sets off a ‘feed forward’ signaling mode, enhancing the inflammatory cascade reaction and subsequently upregulating RAGE expression [216]. Efficient removal of AGEs, downregulation of RAGE expression or other inhibitory approaches targeting the AGE–RAGE axis may play critical roles in managing inflammation-related complications of diabetes, particularly in cases involving endothelial cell inflammation associated with vascular injury and sustained inflammatory reaction hindering wound healing in foot diseases [217–219]. For instance, miRNA-221-3p transported by EPC-Exos exhibits protective effects during hyperinflammatory reactions by partially downregulating the expression of critical AGE–RAGE signaling cascade proteins [220]. Studies have also explored exosomes engineered with RAGE-binding peptide (RBP), which serve a dual purpose as carriers for anti-inflammatory molecules and as a cytoprotective reagent by blocking the positive feedback loop of RAGE-mediated proinflammatory signal transduction [221, 222]. Kim *et al*. used pulmonary-delivered RBP-Lamp2b-modified exosomes to transport curcumin, an FDA-approved natural drug known for its anti-inflammatory and antioxidant properties, through multiple pathways [222, 223]. Both *in vivo* and *in vitro* assays demonstrated the improved efficiency of RBP-chimeric and curcumin-loaded exosomes in reducing hemolysis and monocyte infiltration. The delivery of exosomes along with curcumin is anticipated to be utilized for managing chronic inflammation in DFUs.

### Re-epithelialization and remodeling

Considering the crucial role of rapid and orderly re-epithelialization in wound closure, targeting the epithelialization phase emerges as a promising therapeutic approach for diabetic wounds. Epidermal tissue regeneration initiates within hours post-injury under favorable conditions [224], marked by the rapid and coordinated proliferation, migration and differentiation of keratinocytes [225]. Fibroblasts also contribute significantly throughout the wound closure and ECM remodeling process, participating in fibrin clot degradation, granulation tissue formation and collagen deposition, and wound contraction through myofibroblast differentiation [226]. However, a high-glucose environment has been shown to hinder re-epithelialization by impairing the migration of both keratinocytes and fibroblasts [34, 35]. While various growth factors like epidermal growth factor [227], fibroblast growth factor (FGF) [228] and granulocyte-macrophage colony-stimulating factor [229], have been developed for diabetic wound treatment, their therapeutic efficiency is often limited, possibly due to their short half-life and alteration of downstream signal pathways influenced by the cellular and molecular heterogeneity of wounded tissue [230, 231]. In this context, exosomes offer an advantageous platform for encapsulating therapeutic growth factors, given their ability to carry diverse cargo and protect it from enzymatic degradation. Exosomes derived from platelet-rich plasma (PRP) are particularly attractive as they are rich in endogenous growth factors secreted by platelets and inherit the regenerative potential of PRP [232, 233]. PRP-Exos act on the transforming growth factor-β (TGF-β) signaling pathway, a key regulator of fibroblast activation and collagen deposition critical for tissue repair. This presents a therapeutic target for ECM remodeling and wound re-epithelialization [234, 235]. Recent trials have demonstrated the effectiveness of TGF-β containing PRP-Exos in promoting epithelial differentiation and collagen synthesis when delivered via fibrin sealant-based sustained-release systems, showcasing the potential of preserving TGF-β bioactivity in lyophilized exosome product [232].

Engineered exosomes can also be exploited to promote the efficient endogenous secretion of growth factors. Intriguingly, several miRNAs that are dysregulated in diabetic wounds, such as miR-155, have been found to either positively or negatively influence wound healing by impacting factors like FGF levels, inflammation, collagen deposition and re-epithelialization [236, 237]. In a study conducted by Gondaliya *et al*., MSC-derived exosomes loaded with an miR-155 inhibitor were used to restore FGF-7 levels and enhance keratinocyte migration, which were negatively regulated by miR-155 *in vitro* [238]. The combination of miR-155 inhibitor and MSC-Exos also significantly improved collagen deposition, angiogenesis and re-epithelialization in an *in vivo* diabetic wound mouse model. In addition, it was demonstrated that peptide RL-QN15 is another effective molecule for the treatment of DFUs, which could promote the expression of vascular endothelial growth factor B and the migration and proliferation of HaCaT cells in a high-glycemic environment [239]. Thus, exosomes loaded with peptide RL-QN15 are expected to promote angiogenesis and re-epithelialization of DFUs with a sustained release of RL-QN15; this combinatorial efficacy has been partially confirmed by the pro-healing potency of mesoporous polydopamine and RL-QN15 nanocomposites [240].

In addition to the vital roles played by keratinocytes and fibroblasts in epithelial regeneration, epidermal stem cells (EpSCs) are essential for maintaining skin cell homeostasis through self-renewal or differentiation, typically migrating upward under normal conditions [241–243]. These specialized EpSCs reside in various compartments of the epidermis, each with its specific microenvironment known as an EpSC niche [244, 245]. Among these compartments, EpSCs from the interfollicular epidermis can be activated to proliferate, migrate and generate distinct suprabasal layers: the stratum spinosum, stratum granulosum and stratum corneum [246]. In a study conducted by Liu *et al*., transfection of human EpSCs with miR-100-5p and intradermal injection of miR-100-5p-enriched ADSC-Exos resulted in increased epidermal thickness and EpSC numbers in the basal layer *in vitro*, with related mechanisms involving myotubularin-related protein 3-mediated elevation of phosphatidylinositol-3,4,5-trisphophate, phosphorylation of AKT and ERK, and upregulation of cyclin D1, E1 and A2 expression [247]. Although these exosome products offer a technically advantageous alternative to stem cell therapies, the inefficiency of exosomal message transfer (i.e. insufficient cargo loading) has limited the validation of their beneficial effects on promoting wound healing in *in vivo* experiments [247]. Therefore, future explorations need to focus on developing and implementing efficient encapsulation strategies to enhance exosome cargo loading, such as utilizing genetically encoded devices reported in a set of EXOsomal Transfer Into Cells devices [82]. Furthermore, apart from stimulating EpSC proliferation endogenously, EpSC-Exos have demonstrated equal efficacy to EpSCs in stimulating impaired diabetic wound healing. This suggests that EpSC-Exos hold promise as a candidate therapeutic agent [248].

### Prospects for treatment of DFU based on engineered exosomes

The chronic nature of DFU presents a significant challenge to conventional treatments, requiring consideration of pathophysiological changes in various body systems and local microenvironmental factors [3]. Addressing such a complex syndrome calls for a holistic treatment approach that covers diverse aspects of DFU pathology. While the therapeutic potential of engineered exosomes in DFU is gaining traction, current research evidence remains limited. However, exosome-based therapies are gradually finding clinical applications in conditions primarily driven by inflammation, such as inflammatory bowel disease and COVID-19 pneumonia [249, 250]. Chronic inflammation serves as a common underlying factor in various aspects of DFU pathogenesis, including DPN, atherosclerotic macrovascular events and the stagnation phase of ulcer healing [39, 251, 252]. Engineered exosomes targeting specific anti-inflammatory signaling pathways (or tissue-repairing signaling) are anticipated to positively impact multiple biological activities involved in diabetic wound healing, whether administered systemically and topically [253]. Homogeneous exosomes delivering multiple agents acting on complementary pathways may enhance therapeutic efficacy. Compared to artificially synthesized nanosystems, exosomes exhibit therapeutic potential comparable to source cells, performing multiple functions in tissue regeneration and ulcer healing. They facilitate ‘proangiogenic plus collagen synthetic’, ‘anti-inflammatory and epithelializing’ or ‘anti-inflammatory and proangiogenic’ roles, among other cascade healing effects. In current research and clinical practice on DFU therapy, MSC-Exos are most extensively studied and have been confirmed to coordinate all stages of the diabetic wound healing process [254]. Furthermore, multifunctional hybrid membrane nanovesicles represent an advancement over exosomes, enabling synchronous execution of overlaid functions from diverse producer cells. These nanovesicles offer promise for developing a highly customizable therapeutic platform for DFU with multiple targeting and diverse therapeutic functions [255].

The efficacy of engineered exosomes for treating DFUs varies depending on whether they are administered topically or systemically. A significant focus of research efforts is on developing various biomaterials as carriers for controlled exosome release, and as macroscopic adjuncts providing single/multiple functions to enhance exosome-based DFU therapy. Hydrogel is the most widely used biological dressing for encapsulating exosomes at wound sites. Its favorable biocompatibility and porous structure allow for extended retention in specific areas and gradual release of tissue-targeting exosomes [256]. Chitosan hydrogel in particular has the added benefit of promoting vascular regeneration and managing dermal infections [257]. In addition, electroconductive hydrogels with soft mechanical properties show promising therapeutic potential by mimicking the electrical transmission properties of natural nerve tissue, thus facilitating axonal regrowth and functional recovery in diabetic neuropathy [159]. Other biomaterials such as human acellular amniotic membrane [258], microneedle patch [259], and nanoscaffolds based on cellulose nanofiber or silk fibroin [260–262] offer unique biological features that sustain the biological activity of exosomes *in vitro*. These materials serve as viable alternatives to hydrogels (for more details see reviews [263, 264]). Notably, human epidermal cells (keratinocytes and fibroblasts) and even smooth muscle cells coming into contact with silk fibroin can stimulate the release of endogenous exosomes conveying significant surpluses of angiogenic and growth factors, which may regulate intercellular communication and promote wound healing together with exogenous exosomes [265–267].

### Challenges and future directions

Nonhealing DFUs are severe complications of both type 1 and type 2 diabetes mellitus, imposing significant financial and medical burdens on patients [268]. This review aims to synthesize fragmented discussions on pathogenetically and microenvironmentally guided exosomal strategies for DFU healing from the existing literature. Thus far, the efficacy of utilizing exosomes alone or in combination with various biomaterials for DFU treatment has shown promise. Apart from their inherent therapeutic properties, exosomes serve as efficient carriers for delivering multiple therapeutic agents, either through engineering on parental cells or direct modification of secreted exosomes. Exosomes with inherent ulcer curative activities have been used to develop a safe and biocompatible drug delivery platform, thereby enhancing treatment effectiveness [11, 12]. Several ongoing clinical trials listed on *ClinicalTrials.gov* are currently evaluating the effectiveness of exosomes in treating refractory cutaneous ulcers or conventional wounds (Table 3).

**Table 3:** Clinical trials of exosomes for wound therapeutics registered on *ClinicalTrials.gov*

ClinicalTrials.gov ** identifier**	**Title**	**Status**	**Conditions**	**Intervention/treatment**	**Phases**	**Year**
NCT02565264	Effect of Plasma Derived Exosomes on Cutaneous Wound Healing	Unknown status	Ulcer	Other: plasma-derived exosomes	Early Phase 1	2015
NCT05475418	Pilot Study of Human Adipose Tissue Derived Exosomes Promoting Wound Healing	Completed	Wounds and injuries	Procedure: adipose tissue derived exosomes	Not Applicable	2022
NCT05243368	Evaluation of Personalized Nutritional Intervention on Wound Healing of Cutaneous Ulcers in Diabetics	Recruiting	Foot, diabetic	Dietary supplement: personalized nutritional intervention	Not applicable	2023
NCT05078385	Safety of Extracellular Vesicles for Burn Wounds	Recruiting	Burns	Drug: AGLE-102	Phase 1	2023

Despite the unique role that engineered exosomes play in DFU healing and the rapid progress in this field, certain inevitable restrictions hinder the clinical transition of such nanosystems from bench to bedside.

(1) The high heterogeneity of exosomes and their overlapping physical and biochemical properties with other EVs, necessitates the development of more intelligent and standardized strategies for effective isolation and sensitive characterization of exosomes [42]. Additionally, standardized preparation protocols are needed to enhance the pharmacokinetic and pharmacodynamic performance of exosome products, ensuring their comparability in efficacy evaluation.
(2) Inefficient exosome secretion and purification techniques results in low yields, limiting their large-scale clinical use [269]. Therefore, developing versatile collection strategies to scale-up exosome production is crucial in this field. One approach involves stimulating producer cells using various methods such as physical signals, molecular interference, environmental factors, external inducers and upregulation of the expression of genes related to enhancing exosome production, which could provide a signal for boosting exosome biogenesis and secretion [82, 270]. Additionally, novel exosome separation methods based on microfluidics and acoustics [271], and ultrafast-isolation systems combining multiple physical methods, show promise in sustaining long-term operation under aseptic conditions, potentially overcoming the limitation of scalability and achieving large-scale exosome manufacturing [272–274].
(3) Despite exhibiting biocompatibility and low immunogenicity as natural delivery vectors, the precise mechanisms of intercellular communication mediated by exosomes remain unclear, posing challenges for safety and efficacy assessment in clinical transformation. Studies have indicated the potential unfavorable consequences of exosome-based therapy, including immunostimulation, immunosuppression and tumor progression [275], which may be linked to the heterogeneous components within exosomes arising from diverse source cells. Therefore, further studies are needed to develop a thorough understanding of exosome action mechanisms and establish necessary clinical efficacy and safety parameters, providing guidance for their application in clinical practice.

While the challenges mentioned above need to be tackled before widespread adoption of engineered exosomes, their versatility and therapeutic potential make them promising strategies to address the complex pathology of DFUs and unravel the ‘black box’ of ulcer healing. In-depth basic and applied research on exosome biogenesis, biological properties, action mechanisms, and associated engineering and characterization strategies is crucial for preclinical development, aiming to minimize side effects and optimize therapeutic benefits in clinical practice. Addressing these scientific questions and technical issues requires multidisciplinary research efforts.

## Conclusions

Comprehensive exploration of the systemic and local pathomechanisms of DFUs has facilitated the development of novel therapeutic modalities. Engineered exosomes, with enhanced curative properties of precise targeting, therapeutic cargo loading, enhanced bioavailability and high yield, have become efficient theranostic platforms for DFUs characterized by complex pathophysiology. They address specific pathological pathways that underlie refractory DFUs, including micro- and macro-angiopathy, peripheral neuropathy, infection, sustained inflammation and re-epithelialization. Although numerous questions are still open prior to large-scale clinical translation of exosome-based therapies, the customization of versatile and efficient exosomal system continues to represent a promising avenue in the treatment of DFUs.

## Abbreviations

ADSCs: Adipose-derived stem cells; AGEs: Advanced glycation end products; AML12 cells: Alpha mouse liver cells; BMSCs: Bone marrow mesenchymal/stem stromal cells; DFU: Diabetic foot ulcer; DPN: diabetic peripheral neuropathy; ECM: Extracellular matrix; EPCs: Endothelial progenitor cells; EpSCs: Epidermal stem cells; EVs: extracellular vesicles; FGF: fibroblast growth factor; HaCaT cells: Human clonal keratinocytes; Lamp2b: Lysosome associated membrane protein-2b; LDL-C: Low-density lipoprotein cholesterol; LDLR: Low-density lipoprotein receptor; miRNA/miR: MicroRNA; MRSA: Methicillin-resistant *S. aureus*; MSCs: Mesenchymal stromal cells; PAD: Peripheral artery disease; PD-L1: programmed death-ligand 1; PRP: Platelet-rich plasma; RAGE: Receptor for AGEs; RBP: RAGE-binding peptide; ROS: Reactive oxygen species; SMSCs: Synovium mesenchymal stem cells; TGF-β: Transforming growth factor beta.
